# Evaluation of two free-living, and one entomopathogenic nematode species (Rhabditida) for controlling *Bactrocera zonata* (Diptera: Tephritidae) in Iraq

**DOI:** 10.2478/jofnem-2025-0001

**Published:** 2025-02-15

**Authors:** Saif Abdulhussein Alghanimi, Ali Asghar Talebi, Reihaneh Gholami Ghavamabad, Majid Pedram

**Affiliations:** Department of Entomology, Faculty of Agriculture, Tarbiat Modares University, Tehran, Iran; Research Institute of Forests and Rangelands, Agricultural Research, Education and Extension Organization (AREEO), Tehran, Iran; Department of Plant Pathology, Faculty of Agriculture, Tarbiat Modares University, Tehran, Iran

**Keywords:** *Acrobeloides*, bioassay, biological control, *Heterorhabditis*, *Oscheius*, peach fruit fly

## Abstract

Fruit flies (Diptera: Tephritidae) are among the most serious agricultural pests globally, causing significant economic losses in fruit production and posing major quarantine concerns for many countries. *Bactrocera zonata*, one of the most harmful species in the family Tephritidae, has recently established in Iraq. Entomopathogenic nematodes (EPNs) and potential EPNs can be used as vital tools in integrated pest management (IPM) programs for both organic and conventional production systems. In our study, three species – two native and free-living, and the other, a native EPN – were isolated from various orchards in Iraq. Their pathogenicity was evaluated in laboratory and greenhouse experiments against the soil-dwelling third-instar larvae of *B. zonata*. In laboratory bioassays, mortality rates varied from 70–98% for *Oscheius myriophilus*, 12–58% for *Acrobeloides saeedi*, and 14–56% for *Heterorhabditis bacteriophora* 12 days post-treatment. The mean lethal concentration (LC_50_) values, measured by infective juveniles (IJs)/larva, were 7.08 for *O. myriophilus*, 104.49 for *A. saeedi*, and 97.74 for *H. bacteriophora* in data set 1. They were 13.50, 86.04, and 86.67 IJs/larva, respectively, in data set 2. These values were determined for third-instar larvae in laboratory tests conducted 12 days post-treatment at 25°C and 60% relative humidity (RH). Under greenhouse conditions, applications of *O. myriophilus*, *H. bacteriophora*, and *A. saeedi* resulted in 50.00–91.75% mortality in fruit tests at a concentration of 250 IJs/larva, and 33.58–67.82% mortality in soil tests against *B. zonata*, at a concentration of 1,000 IJs/larva 19 days after treatment. Our results suggest that two presently studied free-living native nematodes are potential EPNs, and together with native isolate of *Heterorhabditis bacteriophora*, could be integrated into pest management programs against *B. zonata* in Iraqi orchards.

The peach fruit fly, *Bactrocera zonata* (Saunders, 1842) (Diptera: Tephritidae), is a highly destructive, polyphagous and multivoltine pest that attacks over 50 host plants worldwide (White and Elson-Harris, 1992; EPPO, 2005). Adult females deposit their eggs inside fruits, where the emerging larvae feed on the pulp, causing significant damage. This feeding often leads to secondary bacterial and fungal infections, frequently resulting in premature fruit drop. After completing three larval instars, the larvae move into the soil to pupate. Overwintering primarily occurs in either the larval or pupal stages (Baker et al., 2019). Originating from South and Southeast Asia, *B. zonata* has now spread across various parts of Asia, including India, Iran, Iraq, Pakistan, Thailand, and others (EPPO, 2022); Africa (Mosleh et al., 2011); Europe, including Austria and France (Egartner et al., 2019; EPPO, 2022); and the Americas, including California and other parts of North America (White and Elson-Harris, 1992; Papadopoulos et al., 2013). Successful control of this pest is particularly challenging due to the larvae’s habit of feeding inside the fruit and pupating in the soil. Several control strategies for *B. zonata* have been explored, including chemical pesticides (Al-Eryan et al., 2018); the male annihilation technique (MAT) and the bait application technique (BAT) (Delrio and Cocco, 2012); plant leaf extracts as sterilant and oviposition deterrents (Mahmoud and Shoeib, 2008; Ilyas et al., 2017); parasitoids (Dashavant et al., 2018; El-Heneidy et al., 2016; Hosni et al., 2011); predators (Momen et al., 2016; Momen et al., 2018); and entomopathogens (Gul et al., 2015; Soliman et al., 2014).

Several aspects of biological control using entomopathogenic nematodes (EPNs) have recently been discussed by Shapiro Ilan and Lewis in their book (2024). Evidence is now gathering about other groups concerning potential and new group of EPNs (Dillmann et al., 2012; Ye et al., 2018). Various studies have revealed that EPNs are more effective in concealed environments, such as within fruit and soil, compared to open habitats (Koppenhöfer, 2000; Lacey et al., 2000; McGraw and Schlossberg, 2017). This improved effectiveness is likely due to the nematodes’ susceptibility to ultraviolet light and desiccation, which are less problematic in protected environments (Gaugler and Boush, 1978; Glazer, 1992). EPNs have demonstrated effectiveness in infecting various stages of *B. zonata*. Adults can be infected by *Steinernema carpocapsae* (Weiser, 1955); *S. riobrave* Cabanillas, Poinar and Raulston, 1994; and *Heterorhabditis bacteriophora* Poinar, 1976 (Abbas et al., 2016; Soliman et al., 2014). Larvae (before pupation) can be targeted by *S. feltiae* (Filipjev, 1934) and *H. marelatus* Liu and Berry, 1996 (Mahmoud et al., 2016; Saleh et al., 2018). Additionally, *S. scapterisci* Nguyen and Smart, 1990 can infect larvae and pupae at soil depths of up to 6 cm (Sayed et al., 2018). Laboratory bioassays revealed that several EPN species, including *H. bacteriophora*, *H. megidis* Poinar, Jackson and Klein, 1987; *H. indica* Poinar, Karunakar and David, 1992; *S. carpocapsae*, *S. riobrave*, *S. feltiae*, and *S. glaseri* (Steiner, 1929) are lethal to *B. zonata* larvae in both fruit and soil, with *H. bacteriophora* causing the highest mortality rates (Usman et al., 2021).

Sallam et al. (2024) reported LC_50_ values of 794.3, 1063.2, 1249.8, and 1446.8 infective juveniles IJs/ml for *S. carpocapsae* (AII), *S. carpocapsae* (EGAZ10), *H. bacteriophora* and *H. indica*, respectively, three days following treatment against *B. zonata*. Field trials demonstrated that a combination of *S. carpocapsae* and *H. bacteriophora* at 3,000 IJs/ml concentration resulted in 97.5% mortality. Wakil et al. (2022) found that combining entomopathogenic fungi (EPF) and EPNs significantly increased mortality rates compared to when they were applied separately, highlighting their potential in integrated pest management (IPM) strategies for tephritid fruit flies in orchard agro-ecosystems.

Heikal et al. (2024) showed that the pathogenicity of EPNs can be markedly enhanced by gamma radiation. Their study demonstrated that *Steinernema carpocapsae* was more effective against *B. zonata* larvae, and *Heterorhabditis bacteriophora* was more effective against pupae after exposure to gamma radiation. In addition to the well-established EPN genera *Steinernema* Travassos, 1927 and *Heterorhabditis* Poinar, 1976, as already stated, other nematode species have also shown promising potential as biological controlling agents. For example, some species of the genus *Oscheius* Andrássy, 1976 have documented insecticidal properties (Qi-Zhi et al., 2012; Zhang et al., 2012; Pervez et al., 2013; Zhou et al., 2017; Ye et al., 2018; Castro-Ortega et al., 2020; Loulou et al., 2022; Gholami Ghavamabad et al., 2021a, b and 2024). Likewise, members of the genus *Acrobeloides* Cobb, 1924 (Nematoda: Cephalobidae) are associated with insects, and certain species exhibit entomopathogenic capabilities (Azizoglu et al., 2016). Beyond insects, *Acrobeloides* species have also been linked to other organisms, including mollusks, arthropods, and annelids (Grewal et al., 2003). *Acrobeloides nanus* (de Man, 1880) has been specifically infected the natural populations of earthworm cocoons (Kraglund and Ekelund, 2002). Additionally, Baquiran et al. (2013) studied the microbial associations of these nematodes, consistently identifying three bacterial species associated with *Acrobeloides maximus* Thorne, 1925. Salari et al. (2021) identified *Acrobeloides* sp. in Iran as a free-living indigenous species, capable of infecting and rapidly killing the larvae of the leopard moth borer *Zeuzera pyrina* (Linnaeus, 1761) (Lepidoptera: Cossidae), suggesting its potential as a biological control agent.

In Iraq, some studies have been conducted on isolation and identification of indigenous EPN species (Al-Zaidawi et al., 2019; Taher and Hassan, 2023). The effect of EPNs against various insect species have also been the subject of some studies (Sayed and Shairra, 2017; Al-Zaidawi et al., 2020). Despite efforts to control *Bactrocera zonata*, this pest has recently become a significant problem due to its larvae feeding inside the fruit and pupating in the soil in certain regions of the country (Gaduaa and Kareem, 2023; EPPO, 2022). First detected in Iraq in 2017 in sweet orange, pomegranate, pear, and pummelo orchards in northeast of Baghdad, *B. zonata* has since spread across citrus and stone fruit orchards in central and southern Iraq (Khlaywi et al., 2017).

The objectives of this study were to evaluate the efficacy of two indigenous free-living and one *Heterorhabditis* species, collected from various soils in Iraq, against *B. zonata* larvae in laboratory and greenhouse experiments.

## Material and Methods

### Nematode isolates, characterization, and culture

The free-living nematode species *Oscheius myriophilus* (Poinar, 1986) (Accession number PQ270466), the EPN species *Heterorhabditis bacteriophora* (Accession number PQ270460) and the free-living nematode species *Acrobeloides saeedi* Siddiqi, De Ley and Khan, 1992 (Accession number PQ270465) were recovered using the soil baiting method (Akhurst and Bedding, 1975) from Karbala (Latitude: 32.65, Longitude: 44.16, Altitude: 45.69m, E: 421699.435m, N: 3613812.602m); Najaf (Latitude: 31.72, Longitude: 44.59, Altitude: 12.09m, E: 461494.110m, N: 3510283.192m); and Diwaniyah (Latitude: 31.92, Longitude: 44.50, Altitude: 22.48m, E: 453227.036m, N: 3532557.618m) between September 2022 and August 2023. All nematode species were cultured in the last-instar larvae of *Galleria mellonella* Linnaeus, 1758 (Lepidoptera: Pyralidae) in a growth chamber following the procedure described by Kaya and Stock (1997). The IJs of the nematode isolates were collected using White traps (White, 1927) and maintained in tissue culture flasks at 4°C.

### Insect collection and rearing

To establish a colony of *Bactrocera zonata*, fruit samples were collected from several orchards in Hosseinieh town (Karbala province) in 2023. Samplings were performed in winter from citrus trees and in summer from apricot trees. Pest infestation was identified by the presence of scars on fruits caused by oviposition. Infested fruits were transported to the laboratory and placed in 5-liter plastic containers with a 2-cm layer of sterile soil at the bottom to collect pupae. Daily checks were conducted to monitor larval emergence and movement into the soil for pupation. After pupation, the soil was sieved to collect pupae, which were transferred to 9-cm diameter glass containers filled with sterile soil. The containers were placed inside a plastic cage (40 × 25 × 25 cm) with side slits covered with fabric for ventilation, maintained at 25°C and 60% relative humidity (RH). For adult fly nutrition, a mixture of 5 g of yeast extract powder and 15 g of sugar was provided in a 9-cm glass container (Slansky and Scriber, 1985). Fly eggs were collected using modified egg collection containers (Zahan et al., 2015). Eggs were placed on a wet sponge and transferred to a 9-cm Petri dish until larval emergence. The larvae were reared on a diet composed of 500 grams of bran flour, 125 grams of ground sugar, 125 g of yeast, 5 g of sodium benzoate, 5 g of citric acid, and 750 ml of sterile water, mixed for 15 minutes and adjusted to a pH of 3.5 to 4 (Afia, 2007).

A colony of the wax moth *Galleria mellonella* was obtained from the Agricultural Research Office (Baghdad, Iraq) and cultured in the laboratory. The larvae were reared on a diet of wheat flour, yeast powder, beeswax, glycerin, and honey in a growth chamber maintained at 28°C, 60% RH, and a 16:8 h light:dark (L:D) photoperiod.

### Laboratory bioassay

To evaluate the susceptibility of *Bactrocera zonata* last (third)-instar larvae to three recovered species (*Oscheius myriophilus*, *Heterorhabditis bacteriophora* and *Acrobeloides saeedi*), bioassays were conducted using a completely randomized design. Plastic containers (3 × 4.5 × 7 cm) were filled with 15 g of sterile soil with initial soil moisture. Nematode suspensions at different concentrations (10, 30, 60, and 100 IJs/larva) were applied to the soil surface. The control treatment received only sterile distilled water without nematodes. Five third-instar larvae were placed onto the soil in each container, which was then covered with a plastic bag and incubated at 25°C, 60% RH, and 16:8 h L:D photoperiod. Each treatment consisted of 10 containers (with five larvae per container), and the experiment was repeated twice, resulting in 100 larvae per treatment and 1,500 larvae overall. Mortality was assessed at three, seven and 12 day intervals post-treatment by calculating adult emergence, which was defined as the total number of fully-emerged insects relative to the initial number of larvae exposed to the nematodes.

### Greenhouse trial: Efficacy of two free-living, and one EPN species on *Bactrocera zonata* in fruit

This experiment was conducted using a randomized complete block design (RCBD). Peach fruits were disinfected with 5% sodium hypochlorite and placed in plastic trays (9 × 13.5 × 22.5 cm), each containing 400 g layer of sterile soil and 80 ml distilled water to maintain soil moisture. A small hole (50 × 6 mm) was made in each fruit using a 120 × 7 mm hollow metal tube, and 50 third-instar larvae of *B. zonata* were placed into the hole using laboratory forceps. The hole was then covered with a small amount of peach fruit pulp. Nematode suspensions of 250 IJs/larva (165 IJs/cm^2^) were applied to the fruits using a 0.5-liter plastic sprayer, while control fruits were treated with sterile distilled water. Before conducting the experiment, the sprayer was tested to ensure proper release of IJs. The trays were covered with net cloth and secured with rubber bands to prevent insect escape. The trays were kept at 25°C and 16:8 h L:D photoperiod. To prevent nematodes from desiccating, the fruits were lightly misted with a sprayer every 48 hours after treatment.

Considering that adult *B. zonata* emerge 11 to 14 days after treatment, dead insects were counted 19 days post-treatment. Fruits were dissected to determine the total number of insects inside, and the presence of nematodes within the insects was recorded. The soil at the bottom of each tray was sieved to recover any remaining insects, and their infection by nematodes was examined using a stereo microscope. Additionally, at the end of the experiment, after 30 days, the fruits were checked under a stereomicroscope to inspect if the infective juveniles (IJs) inside the fruit were still alive. Each treatment (*Oscheius myriophilus*, *Heterorhabditis bacteriophora*, *Acrobeloides saeedi*, and control) consisted of five trays, each containing four fruits, with 50 third-instar larvae per fruit. The experiment was conducted twice, and thus a total of 8,000 larvae were tested.

### Greenhouse trial: Efficacy of two free-living and one EPN species on *Bactrocera zonata* in soil substrate

Plastic trays (6 × 28.5 × 34.5 cm) were filled with 400 g of sterile soil and moistened with 80 ml of distilled water. Fifty last-instar larvae were placed on the soil surface, and 1,000 IJs/larva (50 IJs/cm^2^) nematode suspensions were sprayed onto the soil using a 0.5-liter plastic sprayer. Control trays received only distilled water. The trays were covered with net cloth and secured with rubber bands to prevent insect escape and kept in greenhouse conditions (25 ± 5°C, 60 ± 10% relative humidity, and a 16:8 h L:D photoperiod). This trial was conducted using a RCBD. The efficacy of the nematodes was determined based on the adult emergence rate 19 days post-treatment. The remaining pupae were dissected to determine whether mortality was caused by nematode infection or by other factors. Each treatment group (two free-living, one EPN species, and the control) consisted of 10 trays, and the experiment was conducted twice, resulting in 20 trays per treatment (1,000 larvae per treatment) and a total of 4,000 larvae tested.

### Data Analysis

Data were analyzed using analysis of variance (ANOVA) in SAS (2002), with insect mortality as the response variable, and the exposure time, IJ concentration, and isolates as the main factors. Mean differences were determined at a significance level of α = 0.05 using Tukey’s test. LC_50_ and LC_90_ values for the nematodes were estimated using Probit analysis in SPSS Statistics 17.0 (IBM, Armonk, New York, USA). Mortality data from the greenhouse tests were adjusted for the control mortality using Abbott’s formula (Abbott, 1925).

## Results

### Laboratory bioassay

**Figure 1:**
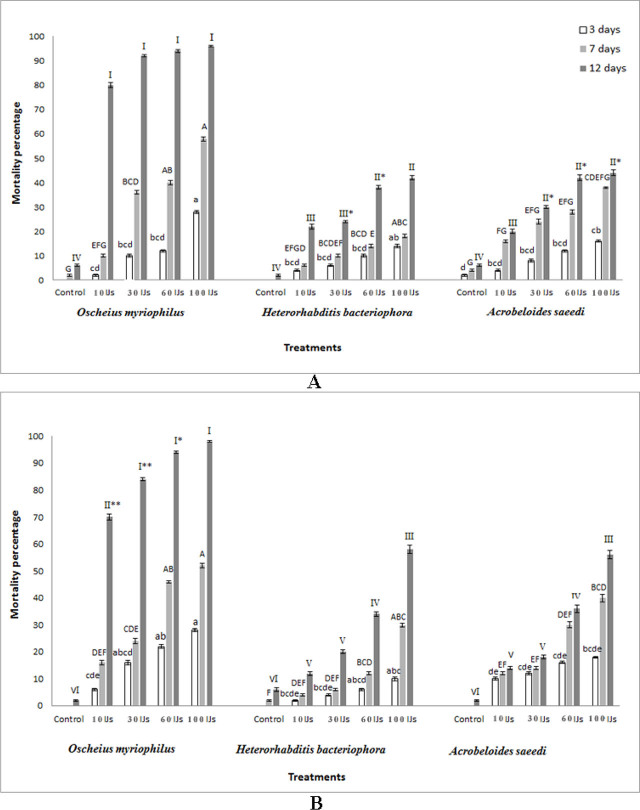
Efficacy of two Iraqi free-living and one EPN species – *Oscheius myriophilus*, *Acrobeloides saeedi* and *Heterorhabditis bacteriophora* – against the last-instar larvae of *Bactrocera zonata* at doses of 0, 10, 30, 60 and 100 IJs/larva at 25°C (Mean ± SE). A. Data set 1 (first trial), B. Data set 2 (repeated trial). Differing lowercase letters indicate significant differences among nematode treatments after three days of exposure (P ≤ 0.05). Differing uppercase letters indicate significant differences among nematode treatments after seven days of exposure (P ≤ 0.05). Differing Latin letters indicate significant differences among nematode treatments after 12 days of exposure (P ≤ 0.05).

In both data sets (the main trial and the repeat), the mortality of *B. zonata* larvae was significantly influenced by nematode species (F = 142.97, df = 2, p < 0.0001; F = 113.15, df = 2, p < 0.0001); nematode concentrations (F = 112.53, df = 4, p < 0.0001; F = 113.46, df = 4, p < 0.0001); and exposure time (F = 277.04, df = 2, p < 0.0001; F = 177.24, df = 2, p < 0.0001). The interaction between nematode species, concentration, and exposure time on mortality was also statistically significant in both data sets (F = 4.22, df = 16, p < 0.0001; F = 3.15, df = 16, p < 0.0001) (Table 1). Across all treatments, mortality rates of larvae consistently increased with IJ concentrations and exposure times (Fig. 1). The highest mortality rates (96% and 98% in data sets 1 and 2, respectively) were recorded with *Oscheius myriophilus* on *B. zonata* at 100 IJs/larva and 12 days post-inoculation.

**Table 1:** Analysis of variance between two Iraqi free-living and one EPN species – *Oscheius myriophilus*, *Acrobeloides saeedi* and *Heterorhabditis bacteriophora* – treatment effects on the third-instar larvae of *Bactrocera zonata* after exposure to dosages 0, 10, 30, 60 and 100 IJs/larva in three, seven and 12-day post-treatment intervals under laboratory bioassays.

	**Source**	**df**	**Sum of squares**	**Mean square**	**F value**	**Pr>F**
Data set 1	Model	44	719.44	16.35	40.50	<0.0001
	Error	405	163.50	0.40	-	-
	Corrected total	449	882.94	-	-	-
	Nematode species	2	115.43	57.71	142.97	<0.0001
	Concentration	4	181.71	45.42	112.53	<0.0001
	Time	2	223.68	111.84	277.04	<0.0001
	Nematode species × Concentration	8	32.43	4.05	10.04	<0.0001
	Nematode species × Time	4	91.40	22.85	56.61	<0.0001
	Concentration × Time	8	47.51	5.93	14.71	<0.0001
	Nematode species × Concentration × Time	16	27.25	1.70	4.22	<0.0001

Data set 2	Model	44	711.49	16.17	31.75	<0.0001
	Error	405	206.30	0.50	-	-
	Corrected total	449	917.79	-	-	-
	Nematode species	2	115.27	57.63	113.15	<0.0001
	Concentration	4	231.18	57.79	113.46	<0.0001
	Time	2	180.56	90.28	177.24	<0.0001
	Nematode species × Concentration	8	36.37	4.54	8.93	<0.0001
	Nematode species × Time	4	70.26	17.56	34.48	<0.0001
	Concentration× Time	8	52.14	6.51	12.80	<0.0001
	Nematode species × Concentration× Time	16	25.69	1.60	3.15	<0.0001

In data set 1, the mortality caused by *O. myriophilus* at concentrations of 10 IJs/larva (80.0%) and 30 IJs/larva (92.0%) was significantly lower than that at 60 IJs/larva (94.0%) and 100 IJs/larva (96.0%) 12 days following treatment. For *H. bacteriophora*, mortality rates at concentrations of 10 IJs/larva, 30 IJs/larva, 60 IJs/larva, and 100 IJs/larva were 20.0, 30.0, 42.0 and 44.0%, respectively, at 12 days after treatment. For the species *A. saeedi*, the mortality percentage in data set 1 was significantly different at 10 IJs/larva (22.0%), 30 IJs/larva (24.0%), 60 IJs/larva (38.0%), and 100 IJs/larva (42.0%) at 12 days after treatment (Fig. 1A). In data set 2, ranges for mortality rates for *O. myriophilus*, *H. bacteriophora*, and *A. saeedi* were 70.0–98.0%, 14.0–56.0%, and 12.0–58.0%, respectively, across concentrations of 10 to 100 IJs/larva, 12 days after treatment (Fig. 1B).

The probit analysis results, and estimated LC_50_ and LC_90_ values at 12 days post-treatment, are summarized in Table 2. The lowest LC_50_ value (7.08 IJs/larva) was determined with *O. myriophilus*, while the highest LC_50_ value (104.49 IJ/larva) was obtained with *A. saeedi* on third-instar larvae of *B. zonata* in the first trial (data set 1).

**Table 2:** LC_50_ and LC_90_ values of two Iraqi free-living, and one EPN species *Oscheius myriophilus*, *Acrobeloides saeedi* and *Heterorhabditis bacteriophora* on the third instar larvae of *Bactrocera zonata* at 12 days post treatment in laboratory bioassay

**Nematode species**		**P-value**	**Slope±SE**	**X^2^ (df=3)**	**Intercept±SE**	**LC_50_**	**LC_90_**
*Oscheius myriophilus*	Data set 1	0.000	0.00±0.00	154.37	−0.21±0.09	7.08 (−8.20–18.16)	49.25 (41.05–57.31)
	Data set 2	0.000	0.00±0.00	136.24	−0.50±0.09	13.50 (−43.01–24.17)	47.62 (23.15–68.56)
*Acrobeloides saeedi*	Data set 1	0.000	0.00±0.00	18.24	−1.14±0.10	104.49 (79.93–167.21)	221.41 (160.42–415.21)
	Data set 2	0.000	0.00±0.00	1.01	−0.50±0.09	86.04 (76.25–99.44)	165.16 (143.35–198.35)
*Heterorhabditis bacteriophora*	Data set 1	0.000	0.00±0.00	13.46	−1.03±0.09	97.74 (87.02–112.50)	218.52 (185.33–263.13)
	Data set 2	0.000	0.00±0.00	6.93	−1.45±0.11	86.67 (66.03–134.60)	162.81 (121.22–289.18)

### Greenhouse trial: Efficacy of two free-living and one EPN species on *Bactrocera zonata* in fruit

**Figure 2:**
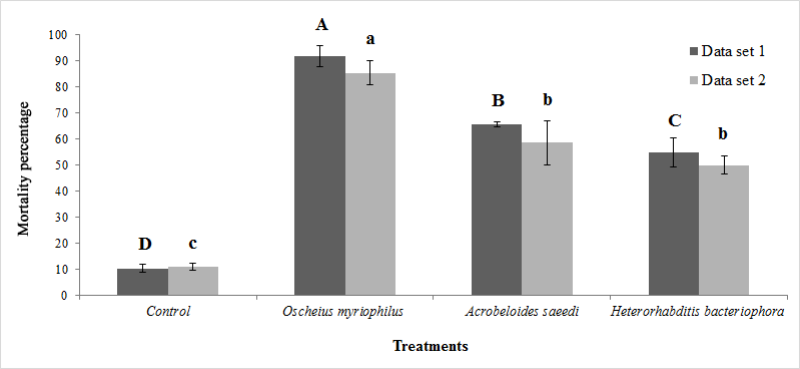
Corrected mortality percentage (Abbott’s formula) of last-instar larvae of *Bactrocera zonata* after exposure to a dose of 250 IJs/larva of two Iraqi free-living and one EPN species – *Oscheius myriophilus*, *Acrobeloides saeedi* and *Heterorhabditis bacteriophora* – 19 days post-treatment in greenhouse trial in fruit (Mean ± SE). Differing uppercase letters indicate significant differences among nematode treatments within data set 1 (P ≤ 0.05). Differing lowercase letters indicate significant differences among nematode treatments within data set 2 (P ≤ 0.05).

In the fruit-based test, *Oscheius myriophilus* yielded the highest larval mortality of *Bactrocera zonata*, with 91.75% in data set 1 and 85.39% in data set 2, respectively, 19 days following treatment at a concentration of 250 IJs/larva (165 IJs/cm^2^). These mortality rates were significantly higher than those caused by *Acrobeloides saeedi,* with 65.66% and 58.65% mortality in data set 1 and data set 2, respectively, and *Heterorhabditis bacteriophora*, with 54.73% and 50.00% mortality in data set 1 and data set 2, respectively (F = 158.26 and F = 67.57 for data set 1 and data set 2, respectively; df = 7, P < 0.0001) (Fig. 2). In data set 2, the mortality rates caused by *A. saeedi* and *H. bacteriophora* were not significantly different. Additionally, no significant differences were found between blocks in the fruit test (F = 0.23, df = 4, P = 0.91 for data set 1; F = 0.57, df = 4, P = 0.69 for data set 2) (Table 3). At the end of the 30-day experiment, IJs were still found alive inside the fruit.

**Table 3:** Analysis of variance results for effects of two Iraqi free-living and one EPN species – *Oscheius myriophilus*, *Acrobeloides saeedi* and *Heterorhabditis bacteriophora* – on the last-instar larvae of *Bactrocera zonata* after exposure to a dose of 250 IJs/larva, 19 days post-treatment, in a greenhouse trial in fruit.

	**Source**	**df**	**Sum of squares**	**Mean square**	**F value**	**Pr>F**
Data set 1	Model	7	17319.07	2474.15	158.26	<0.0001
	Error	12	187.59	15.63	-	-
	Corrected total	19	17506.67	-	-	-
	Treatments (Nematode species)	3	17304.72	5768.24	368.97	<0.0001
	Block	4	14.34	3.58	0.23	0.91

Data set 2	Model	7	14279.14	2039.87	67.57	<0.0001
	Error	12	362.25	30.18	-	-
	Corrected total	19	14641.40	-	-	-
	Treatments (Nematode species)	3	14210.85	4736.95	156.92	<0.0001
	Block	4	68.29	17.07	0.57	0.69

### Greenhouse trial: Efficacy of two free-living and one EPN species on *Bactrocera zonata* in soil substrate

**Figure 3:**
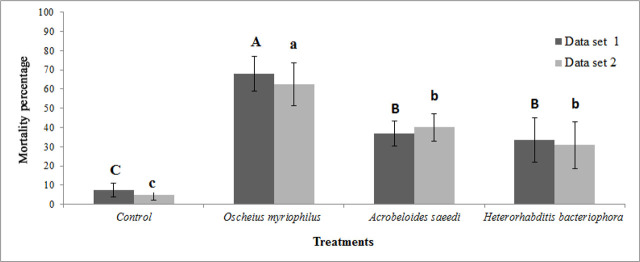
Corrected mortality percentage (using Abbott’s formula) of last-instar larvae of *Bactrocera zonata* after exposure to a dose of 1,000 IJs/larva of two Iraqi free-living and one EPN species – *Oscheius myriophilus*, *Acrobeloides saeedi* and *Heterorhabditis bacteriophora –* 19 days post-treatment, in greenhouse trial in soil substrate (Mean ± SE). Differing uppercase letters indicate significant differences among nematode treatments within data set 1 (P ≤ 0.05). Differing lowercase letters indicate significant differences among nematode treatments within data set 2 (P ≤ 0.05).

Significant differences were observed among treatments in soil-based tests (F = 28.75 and F = 14.86 for data set 1 and data set 2, respectively; df = 12, P < 0.0001). All nematode treatments resulted in fewer emerging adult flies compared to the control (Fig. 2). *Oscheius myriophilus* was substantially more effective than other treatments, yielding in 67.82% and 62.53% larval mortality of *Bactrocera zonata* in data set 1 and data set 2, respectively, 19 days post-treatment at a concentration of 1,000 IJs/larva (50 IJs/cm^2^). These mortality rates were significantly higher than those caused by *Acrobeloides saeedi* (36.72% and 40% mortality in data set 1 and data set 2, respectively) and *Heterorhabditis bacteriophora* (33.58% and 30.94% mortality in data set 1 and data set 2, respectively) (P < 0.0001) (Fig. 3). Mortality rates caused by *A. saeedi* and *H. bacteriophora* were not significantly different in both data set 1 and 2. No significant differences were found between blocks in the soil tests (F = 1.83, df = 9, P = 0.10 for data set 1; F = 0.39, df = 9, P = 0.92 for data set 2) (Table 4).

**Table 4:** Analysis of variance results of two Iraqi free-living and one EPN species – *Oscheius myriophilus*, *Acrobeloides saeedi* and *Heterorhabditis bacteriophora* – on the last-instar larvae of *Bactrocera zonata* after exposure to a dose of 1,000 IJs/larva, 19 days post-treatment, in a greenhouse trial, in soil substrate.

	**Source**	**df**	**Sum of squares**	**Mean square**	**F value**	**Pr>F**
Data set 1	Model	12	19282.74	1606.89	28.75	<0.0001
	Error	27	1509.15	55.89	-	-
	Corrected total	39	20791.89	-	-	-
	Treatments (Nematode species)	3	18361.48	6120.49	109.50	<0.0001
	Block	9	921.26	102.36	1.83	0.10

Data set 2	Model	12	17330.06	1444.17	14.86	<0.0001
	Error	27	2624.65	-	-	-
	Corrected total	39	19954.72	-	-	-
	Treatments (Nematode species)	3	16985.17	5661.72	58.24	<0.0001
	Block	9	344.89	38.32	0.39	0.92

## Discussion

*Bactrocera zonata* represents a suitable target for control using well-accepted and potential EPNs due to its hidden habitat. Our laboratory bioassays demonstrated that *O. myriophilus* was more effective than *H. bacteriophora* and *A. saeedi* against *B. zonata* larvae. At a concentration of 100 IJs/larva, *O. myriophilus* yielded mortality rates ranging from 28.0% to 98.0% in third-instar larvae in the three to 12 days post-treatment. Similarly, our greenhouse tests confirmed the efficacy of three studied nematode species in controlling *B. zonata* larvae in both fruit and soil environments. *Oscheius myriophilus* was, again, more effective than the other two nematode species, causing 85.39% to 91.75% mortality in the fruit tests and 62.53% to 67.82% mortality in the soil tests 19 days after treatment. By comparison, *A. saeedi* resulted in 58.65% to 65.66% mortality in the fruit tests and 36.72% to 40.0% mortality in the soil tests, while *H. bacteriophora* caused 50.0% to 54.73% mortality in the fruit tests and 30.94% to 33.58% mortality in the soil tests 19 days after treatment. These findings align with previous research by Sallam et al. (2024), who reported 10.0% and 35.0% mortality of last-instar larvae of *B. zonata* at concentrations of 250 and 500 IJs/ml of *H. bacteriophora*, respectively, in a laboratory test three days after treatment. This is comparable to our results, where mortality ranged from 10.0% to 18.0% at similar concentrations and time intervals. Langford et al. (2014) found that exposing *Bactrocera tryoni* (Froggatt, 1897) (Diptera: Tephritidae) to 500 IJs/cm^2^ of *H. bacteriophora* in a soil test resulted in 50.0% mortality after 14 days, similar to our observed mortality rates, which ranged between 44.0% and 56.0% in *B. zonata* larvae after 19 days. Yasir et al. (2023) recorded mortality rates between 39.5% and 86.7% for *B. zonata* larvae treated with *H. bacteriophora* at concentrations of 100 to 400 IJs/ml in Iraq, which were higher than our results. Usman et al. (2021), reported 95.74% mortality in *B. zonata* larvae at 5,000 IJs/ml of *H. bacteriophora* in a potted-soil bioassay, with the lowest adult emergence (19%) observed in *H. bacteriophora*-treated larvae and pupae under greenhouse conditions. Interestingly, in our study, *O. myriophilus* outperformed *H. bacteriophora*, a species commonly utilized in other studies.

To date, the efficacy of free-living nematodes from the genera *Oscheius* and *Acrobeloides* on *B. zonata* have not been studied. However, their efficacy against other pests has been documented. For instance, *Oscheius tipulae* (Lam and Webster, 1971) has been shown approximately 65.0% and 75.0% mortality in *Ceratitis capitata* (Wiedemann, 1824) (Diptera: Tephritidae) when applied at concentrations of 250 and 500 IJs per larva (Loulou et al., 2022). In another study, *Oscheius* sp. successfully infected and killed 30.0% of late-third-instar larvae of *Anastrepha fraterculus* (Wied., 1830) (Diptera: Tephritidae) at 100 IJs/larva under laboratory conditions (25 ± 2ºC, RH 70 ± 10%) (Foelkel et al., 2016). In our study, *O. myriophilus* achieved mortality rates of 94.0% to 98.0% at similar concentrations under laboratory conditions, demonstrating high potential for controlling *B. zonata*.

The present study highlights the potential of native nematodes, particularly *O. myriophilus*, for managing *B. zonata* in both greenhouse and laboratory settings. Several factors, such as humidity, soil type, environmental conditions, and application methods, can influence the effectiveness of EPNs and potential EPNs. In field conditions, where agricultural systems are more complex, these factors can alter outcomes. Infective juveniles can be applied using various methods, including backpack sprayers, boom sprayers, trunk sprayers, subsurface injection (Toledo et al., 2023), drip irrigation systems (Erdoğan and Ulu, 2024), robotic system (Erdoğan et al., 2021; 2023) or capsule techniques (Ulu & Erdoğan, 2023). However, it is also essential to understand the physical and chemical properties of the soil in fruit orchards in order to optimize IJ performance.

As already discussed, specific criteria must be met for a nematode isolate to be considered an EPN (Dillman et al., 2012). These criteria have now been fulfilled in the case of two *Oscheius* spp. (Dillman et al., 2012). Moreover, new EPNs continue to emerge in various detailed biological evaluations. Although not all parameters as defined by Dillmann et al. (2012) were met, most of these criteria were satisfied for the two nematode species studied here, *Oscheius myriophilus* and *Acrobeloides saeedi*. Based on the results presented, these species/isolates could be regarded as new potential EPNs. However, further studies are required to evaluate all six prerequisite criteria, which the authors plan to address in a future independent study.

Future research should focus on large-scale field applications of EPNs and potential EPNs to validate these findings under real-world circumstances. Additionally, further studies should explore the post-inoculation biology of these nematodes, their interaction with environmental factors, and their potential synergistic effects when combined with other microbial control agents or integrated pest management strategies. These insights could lead to more effective and sustainable pest management strategies.
